# Nanocrystallography measurements of early stage synthetic malaria pigment

**DOI:** 10.1107/S1600576717012663

**Published:** 2017-09-28

**Authors:** Ruben A. Dilanian, Victor Streltsov, Hannah D. Coughlan, Harry M. Quiney, Andrew V. Martin, Nectarios Klonis, Con Dogovski, Sébastien Boutet, Marc Messerschmidt, Garth J. Williams, Sophie Williams, Nicholas W. Phillips, Keith A. Nugent, Leann Tilley, Brian Abbey

**Affiliations:** a ARC Centre of Excellence in Advanced Molecular Imaging, School of Physics, The University of Melbourne, Melbourne, Victoria 3010, Australia; b CSIRO, Parkville, Victoria 3052, Australia; c ARC Centre of Excellence in Advanced Molecular Imaging, Department of Chemistry and Physics, La Trobe Institute for Molecular Science, La Trobe University, Melbourne, Victoria 3086, Australia; d The Walter and Eliza Hall Institute of Medical Research, Parkville, Victoria 3052, Australia; e CSIRO Manufacturing Flagship, Parkville, Victoria, Australia; f Department of Biochemistry and Molecular Biology, Bio21 Molecular Science and Biotechnology Institute, The University of Melbourne, Melbourne, Victoria 3010, Australia; g LiNAC Coherent Light Source, SLAC National Accelerator Laboratory, Menlo Park, CA 94025, USA; h BioXFEL STC, 700 Ellicott Street, Buffalo, NY 1420, USA; i Brookhaven National Laboratory, PO Box 5000, Upton, NY 11973-5000, USA

**Keywords:** crystallography, serial femtosecond nanocrystallography, malaria, crystalline disorder, structural inhomogeniety

## Abstract

The recent availability of extremely intense, femtosecond X-ray free-electron laser (XFEL) sources has spurred the development of serial femtosecond nanocrystallography (SFX). Here, SFX is used to analyze nanoscale crystals of β-hematin, the synthetic form of hemozoin which is a waste by-product of the malaria parasite. This analysis reveals significant differences in β-hematin data collected during SFX and synchrotron crystallography experiments. To interpret these differences two possibilities are considered: structural differences between the nanocrystal and larger crystalline forms of β-hematin, and radiation damage. Simulation studies show that structural inhomogeneity appears at present to provide a better fit to the experimental data. If confirmed, these observations will have implications for designing compounds that inhibit hemozoin formation and suggest that, for some systems at least, additional information may be gained by comparing structures obtained from nanocrystals and macroscopic crystals of the same molecule.

## Abstract 2

Serial femtosecond crystallography (SFX) at an X-ray free-electron laser enables crystllographic data to be collected from samples orders of magnitude smaller than at a synchrotron. Here SFX is used to investigate the nascent structure of β-hematin derived from nanocrystals and this is compared with the well known structure derived from macroscopic crystals of the same material.

## 1. Introduction   

*Plasmodium falciparum* is the causative agent of the most severe form of malaria in humans. During the blood stage of its lifecycle the malaria parasite invades red blood cells. It develops through the early (ring) stage to the mature (trophozoite) stage and then divides in the schizont stage. As it grows, the parasite digests hemoglobin, producing hematin – ferroprotoporphyrin IX (FP) – as a by-product. FP is a toxic molecule that can damage membranes and proteins (Balla *et al.*, 2007 ▸; Becker *et al.*, 2004 ▸; Kumar & Bandyopadhyay, 2005 ▸). To avoid these toxic effects the parasite sequesters FP into non-reactive crystals, known as hemozoin or malaria pigment. Quinoline and quinoline-related antimalarials target the parasite by preventing the formation of hemozoin crystals, leading to poisoning of the malaria parasite (Egan, 2001 ▸; Klonis *et al.*, 2013 ▸; Slater & Cerami, 1992 ▸; Tilley *et al.*, 2001 ▸). Efforts to design improved quinolines require a detailed understanding of the process of formation of hemozoin, and the structural properties of the initiating crystals. Some members of this drug class, such as chloroquine, have been rendered ineffective by the development of resistance by the parasite, but others, such as mefloquine and lumefantrine, are still in use.

Hemozoin is isostructural with a synthetic phase, β-hematin, whose crystal structure has previously been determined by X-ray powder diffraction (XRPD) (Pagola *et al.*, 2000 ▸). The structures of hemozoin and β-hematin are very similar (Bohle *et al.*, 1997 ▸; Pagola *et al.*, 2000 ▸). However, there is increased heterogeneity in the Fe—O coordination in hemozoin, which leads to a greater disorder in the crystal packing compared with β-hematin (Klonis *et al.*, 2010 ▸). The unit cell of the β-hematin crystal consists of two FP molecules, connected *via* two reciprocal Fe—O bonds between the Fe atom of one molecule and one of the propionate side chains, CH_3_–CH_2_–C(⋯O)—O, of the other molecule. The resulting FP dimers are further stabilized by hydrogen-bonding interactions involving the remaining propionate side chains and by π–π interactions between the non-chelated porphyrin faces of the FP molecules (Klonis *et al.*, 2010 ▸). Assays of inhibition of β-hematin formation *in vitro* are widely used as a convenient initial screen in the search for new antimalarial drugs.

In the mature (trophozoite) stage of parasite development, crystal growth is assumed to involve the addition of FP dimers onto the growing faces of pre-existing hemozoin crystals (Pisciotta & Sullivan, 2008 ▸; Egan, 2008 ▸; Chugh *et al.*, 2013 ▸; Bendrat *et al.*, 1995 ▸). Quinolines are thought to exert their activity by binding to free hematin on to the growing face of the macroscopic crystals (Weissbuch & Leiserowitz, 2008 ▸; Combrinck *et al.*, 2013 ▸), which prevents hematin sequestration and eventually kills the parasite.

Much less is known about the structure of hemozoin nanocrystals (Pisciotta *et al.*, 2007 ▸; Kapishnikov *et al.*, 2012 ▸). Hemoglobin digestion begins when the parasites are morphologically at the ring stage of development (Gruring & Spielmann, 2012 ▸; Abu Bakar *et al.*, 2010 ▸). During the early stages of crystallization the initiating nanocrystals might be expected to present different surfaces from the larger crystals. This has important implications for the development of any compounds that target these nascent structures because antimalarial therapeutics designed on the basis of structural knowledge derived from larger crystals may be less effective against the early stage of intraerythrocytic development. Any differences observed in the nanocrystal and macroscopic crystal structures may have direct consequences for providing prompt therapy for severe malaria infections. The desire to limit the size of β-hematin crystals formed as a by-product of the malaria parasite has motivated the present XRPD studies of nanoscale (<1 µm) β-hematin crystals.

## 2. Results   

### 2.1. XRPD experiments at the synchrotron and X-ray free-electron laser   

X-ray diffraction data were collected at the macromolecular crystallography beamlines, MX1 and MX2, at the Australian Synchrotron and at the Linac Coherent Light Source (LCLS) X-ray free-electron laser (XFEL). At the XFEL, data were collected using the serial femtosecond crystallography (SFX) technique (Darmanin *et al.*, 2016 ▸; Chapman *et al.*, 2011 ▸; Barends *et al.*, 2014 ▸), in which small crystals are illuminated by extremely bright femtosecond X-ray pulses. The data were then assembled into a form that emulates a set of powder diffraction data. The SFX data were obtained with a beam size of 1 × 1 µm full width at half-maximum (FWHM). The synchrotron beam size was 130 × 90 µm (MX1) and 37 × 32 µm (MX2) FWHM. The corresponding doses, calculated using *RADDOSE* (Zeldin *et al.*, 2013 ▸), were 0.26 MGy (MX1) and 9.21 MGy (MX2). The diffraction patterns in all three experiments were measured from crystals derived from the same β-hematin sample. It is important to note that, in general, even small β-hematin crystals are polycrystalline; throughout this paper we refer to the smaller subunits (or ‘grains’) as ‘crystallites’ and the individual β-hematin polycrystals themselves simply as ‘crystals’. In the SFX and MX2 cases the crystals were passed through a filter which allowed through only sub-micrometre crystals. For heterogeneous samples the difference in beam size at the synchrotron *versus* the XFEL has the effect that the larger crystals dominate the diffraction signal over the smaller crystals. In the MX1 data from an unfiltered sample where the structural data closely match the published structure for β-hematin (Pagola *et al.*, 2000 ▸), we can therefore assume that it is mainly the larger crystals that dominate the diffraction. In the case of the MX2 data, however, where the crystals were subject to the same filtration as at the XFEL, the average crystal size is reduced and much more homogeneous: though unlike at the XFEL the very smallest crystals still cannot contribute significantly to the diffraction.

**Figure 1:**
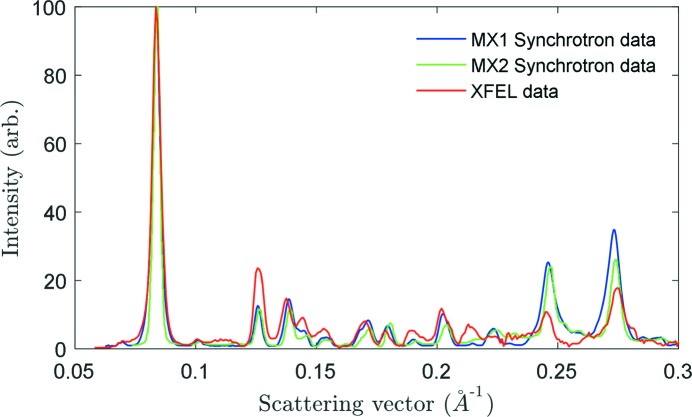
Synchrotron MX1 unfiltered crystal data (blue) and MX2 filtered crystal data (green) and SFX XRPD pattern (red) from β-hematin.

Powder diffraction patterns obtained from both SFX and synchrotron experiments are shown in Fig. 1 ▸. Analysis of the diffraction data reveals a significant difference between the XRPD patterns obtained from the different experiments. Whilst the MX1 synchrotron data are consistent with the published β-hematin diffraction data (Bohle *et al.*, 1997 ▸; Pagola *et al.*, 2000 ▸) (also see supplementary information), the MX2 synchrotron data show some minor deviations and the SFX XRPD data some very significant differences from the previously published data. In particular, the ratios of peak intensities of pairs of Bragg reflections 001 and 020, 031 and 131 do not coincide with the previously published values (Table S1). The greater similarity between the MX1 and MX2 data compared to the XFEL data arises because, even after filtering, the sample consists of crystals that range in size from ∼50 nm up the largest size allowed through by the filter, ∼1 µm. At MX2 the beam size is much closer to that of MX1 than to the XFEL and also has a greatly reduced intensity compared to the XFEL. This means that a large portion of the filtered material will be too small to contribute significantly to the measured powder pattern. By contrast, at the XFEL almost every crystal gave a measurable signal (‘hit rates’ were above 90%), from the smallest nanocrystals up the largest 1 µm fragments. Hence we expect that the effect will be much more subtle between synchrotron experiments than between synchrotron and XFEL experiments.

The SFX XRPD pattern exhibits a much stronger scattering of X-rays within the 0.21–0.22 Å^−1^ range, as well as a significant reduction of the intensities of the 031 and 131 Bragg reflections, which are usually much stronger than the 001 Bragg reflection. This diffraction pattern for β-hematin has not been reported in any previous study (Straasø *et al.*, 2011 ▸, 2014 ▸; Solomonov *et al.*, 2007 ▸; Pagola *et al.*, 2000 ▸; Klonis *et al.*, 2010 ▸; Bohle *et al.*, 1997 ▸). In the discussion that follows we examine the possible origin for the significant differences between the measured SFX and synchrotron XRPD patterns.

#### 2.1.1. Cryogenic *versus* room temperature   

The first issue to consider is the fact that the synchrotron samples were measured at cryogenic temperatures whereas the XFEL data were not. Structural studies of β-hematin conducted both under cryogenic conditions and at room temperature have been previously published. As noted by Bohle *et al.* (1997 ▸), when the results from the cryogenic experiments are compared with structural data collected at room temperature only very minor differences are observed (the changes we observe are much more dramatic). Whilst this may seem surprising, β-hematin is a small molecule and quite different from a protein macromolecule (whose crystals typically contain greater than 50% water by weight). On the basis of published data we can therefore rule out temperature as a possible explanation for the observed differences in the data.

#### 2.1.2. Filtering-induced stress and nonlinear detector response   

Another point to note is whether stress could have been introduced into the β-hematin crystals during filtering. β-Hematin is an organometallic complex with an Fe atom contained in the centre of a heterocyclic porphyrin ring, and made of much more robust material than typical protein crystals. It is very unlikely, therefore, that filtering of a solid and robust polycrystalline material could induce significant stress. Filtering is also used routinely for size separation of β-hematin crystals in the literature without any reports of significant damage to the structure being observed.

We have also considered the nonlinear response of the Cornell–SLAC pixel array detector (CSPAD) used at the XFEL and whether this could account for the differences between the SFX and synchrotron XRPD patterns. The complex nonlinear signal response from a pixel array detector is reviewed in detail by van Driel *et al.* (2015 ▸). A discussion of the treatment of data presented here, prior to analysis, is given in the supplementary information. Whilst the SFX data taken at the XFEL do not show indications of saturation, there are signs of nonlinearity in the raw data (see Fig. S3), though we note these effects are generally below the observed signal changes.

We also note that filtering-induced stress and nonlinear detector responses, as well as radiation damage effects, are not consistent with the observation that not all peaks decrease in relative intensity when comparing XFEL and synchrotron data. For example there is stronger scattering of X-rays within the 0.21–0.22 Å^−1^ range, and the 001 peak actually becomes stronger. These observations do not support the theory that the significant differences observed in the data could be explained by the first two factors. Structural differences between crystals of different size or the effects of radiation damage, however, could potentially account for these observations in both the XFEL and synchrotron data. We now turn our discussion to an investigation of these effects and whether they are able to reproduce the measured diffraction data.

### 2.2. Radiation damage   

The effect of radiation damage in the context of XFELs can be to suppress the contribution of the higher-resolution diffraction peaks. This topic has been explored both in simulation and in experiment by Barty *et al.* (2012 ▸). In addition, a recent experimental paper by Nass *et al.* (2015 ▸) has looked at the effects of XFEL-induced photoreduction of Fe in ferredoxin.

For the synchrotron experiments, the doses for MX1 and MX2 were 0.26 and 9.21 MGy, respectively. Although these numbers are different, they are both well below the Henderson absorption limit of 20 MGy (Henderson, 1990 ▸). Local radiation damage in the form of photoreduction of Fe has similarly not been observed during previous synchrotron X-ray absorption spectroscopy studies when using a dose comparable to or greater than that in the current study (Kuter *et al.*, 2016 ▸; Gildenhuys *et al.*, 2015 ▸).

In the context of radiation damage at the synchrotron, both cryogenic temperatures and continuous scanning of the sample were used to mitigate the effects of global damage. There is also an extremely good match between the MX1 synchrotron data and the published β-hematin structure, which, assuming the published structure is not itself modified by radiation damage, gives confidence that this effect was minimal in the present case. In addition, when we compare the data set collected at the MX2 beamline from crystals subjected to the same filtration as at the XFEL we see some of the same characteristic differences in the data that are so pronounced at the XFEL.

For the SFX experiments, simulations of the peak attenuation for β-hematin (see Fig. S4) show a small suppression of the high-resolution peaks due to global radiation damage at the XFEL. Another effect that can modify the peak intensity is the isotropic displacement factors. This will result in suppression of all peak intensities, similar to global damage. Whilst global damage may explain some of the reduction of the 031 and 131 peaks, it does not account (for example) for the increase in intensity of the 001 peak. The closer proximity to the Fe edge makes local damage more likely at the XFEL compared to the synchrotron. Fe^III^ can be photoreduced to Fe^II^ or even further and could produce a relative increase in certain reflections, since the molecular structure itself has changed locally. Nass *et al.* (2015 ▸) explored this effect using a 200 × 200 nm focused beam, 80 fs pulse duration, with an energy tuned 0.25 keV above the Fe edge (7.11 keV).

Under the conditions used here (1 × 1 µm beam, 30 fs pulse duration, 1.4 keV above the Fe *K* edge), the effects of photoreduction of Fe^III^ are expected to be smaller compared to the work of Nass *et al.*, who also used an incident energy tuned to the Fe *K* edge. To explore this effect, we carried out a series of simulations, replacing the Fe atoms with different elements having a reduced number of electrons. These simulations are based on the ideal crystal structures without the temperature factors included (which add to global damage), but are sufficient to gauge the magnitude of the local damage effect on peak ratios. The magnitude of the local damage effect in these systems was found, in these simple simulations, to be too small to account for the observed differences. However, the combination of global and local radiation damage can modify the diffraction intensities in complex ways that are not captured in the simulations performed here. In fact, currently, the interplay of local and global radiation damage effects is a topic of active research within XFEL science. Hence it is impossible to entirely discount this as a potential explanation for the observed differences between the synchrotron and XFEL experiments.

A final point to consider is electronic motion which can occur on femtosecond timescales. Whilst modification of the diffraction intensities has been observed in the case of C_60_ nanocrystals owing to electronic rearrangement during interaction with an XFEL pulse (Abbey *et al.*, 2016 ▸; Ryan *et al.*, 2017 ▸), the incident X-ray flux in the present case is significantly lower (see 4§[Sec sec4]). In addition the high degree of symmetry of C_60_ molecules facilitates long-range correlations of the electronic structure on a femtosecond timescale.

### 2.3. Structural inhomogeneity in nanocrystals   

The wavelength region of the XRPD patterns where such diffracted intensity variations are observed corresponds to the low- to middle-level resolution range, 11.0–3.0 Å. This length scale indicates that the changes arise from rearrangement of the FP molecules in the crystalline lattice. As an alternative to the radiation damage theory, we reanalyzed the known structure of β-hematin (Pagola *et al.*, 2000 ▸) in terms of spatial alignment of the FP molecules. Analysis conducted in this manner allows us to identify simple relationships between the intensity ratios of selected Bragg reflections and the spatial configuration of the FP dimers as well as the curvature of the FP molecules.

**Figure 2:**
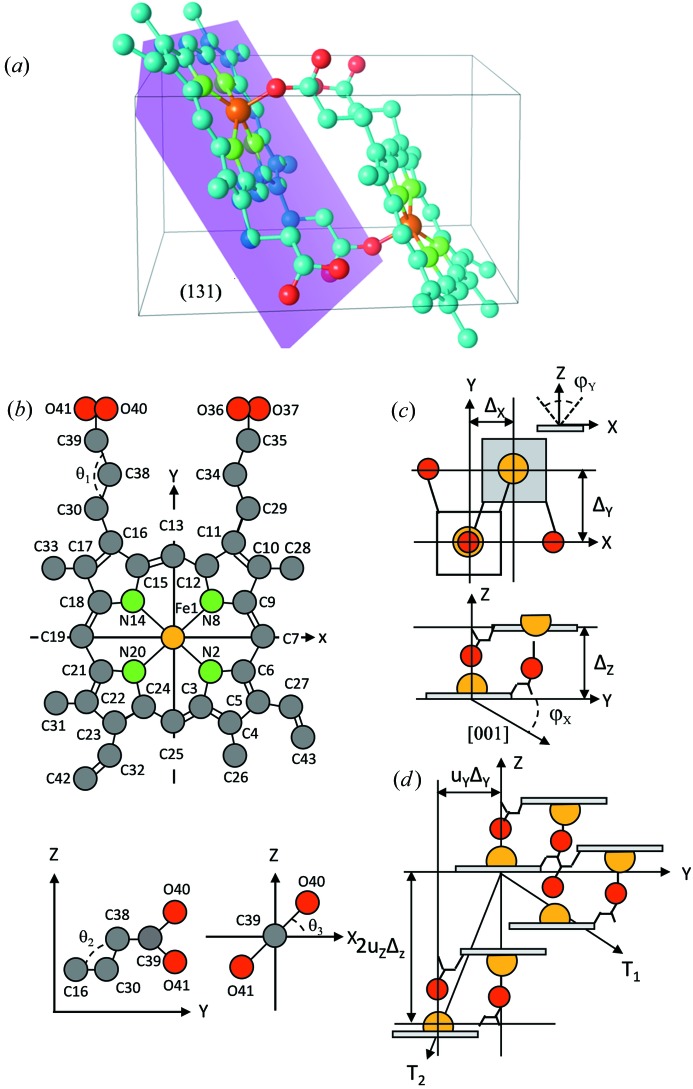
(*a*) Crystal structure of β-hematin. The molecules stack parallel to the (131) crystallographic plane. (*b*) Model of the β-hematin molecule corresponding to (*a*), showing angles 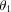 (top), 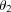 and 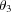 (bottom). (*c*) Schematic representation of the formation of the FP dimer. The orientations of the porphyrin ring about the *xy* and *xz* planes are described by 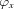 and 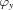, respectively. (*d*) The two-dimensional layers of the FP dimers.

For convenience, we consider the structure of the FP molecule (see Fig. 2 ▸ *a*) in an orthogonal system, assuming that the porphyrin ring of the molecule is located in the *xy* plane (Fig. 2 ▸ *b*). Given the orientation the Fe—O bonds, which are normal to the porphyrin ring planes, and the rigid structure of the porphyrin ring of the FP molecule, the relative displacement of two FP molecules forming an FP dimer (Figs. 2 ▸ *c* and 2 ▸ *d*) can be described by the displacement vector 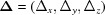, where Δ*_x_*, Δ*_y_* and Δ_*z*_ are defined in Fig. 2 ▸. The relative displacement of the molecules is defined by the relative displacement of the Fe atom from the porphyrin ring plane and by the position of the O atom. The latter is influenced by the conformation of the propionate side chain, especially by the θ_1_, θ_2_ and θ_3_ angles (Fig. 2 ▸ *b*). The key result here is that the form of the FP dimer depends upon the conformational flexibility of the propionate groups which permits the formation of the Fe—O and O—H⋯O bonds between the nearest FP molecules. This in turn modifies the crystal structure and hence the measured β-hematin diffraction pattern.

**Figure 3:**
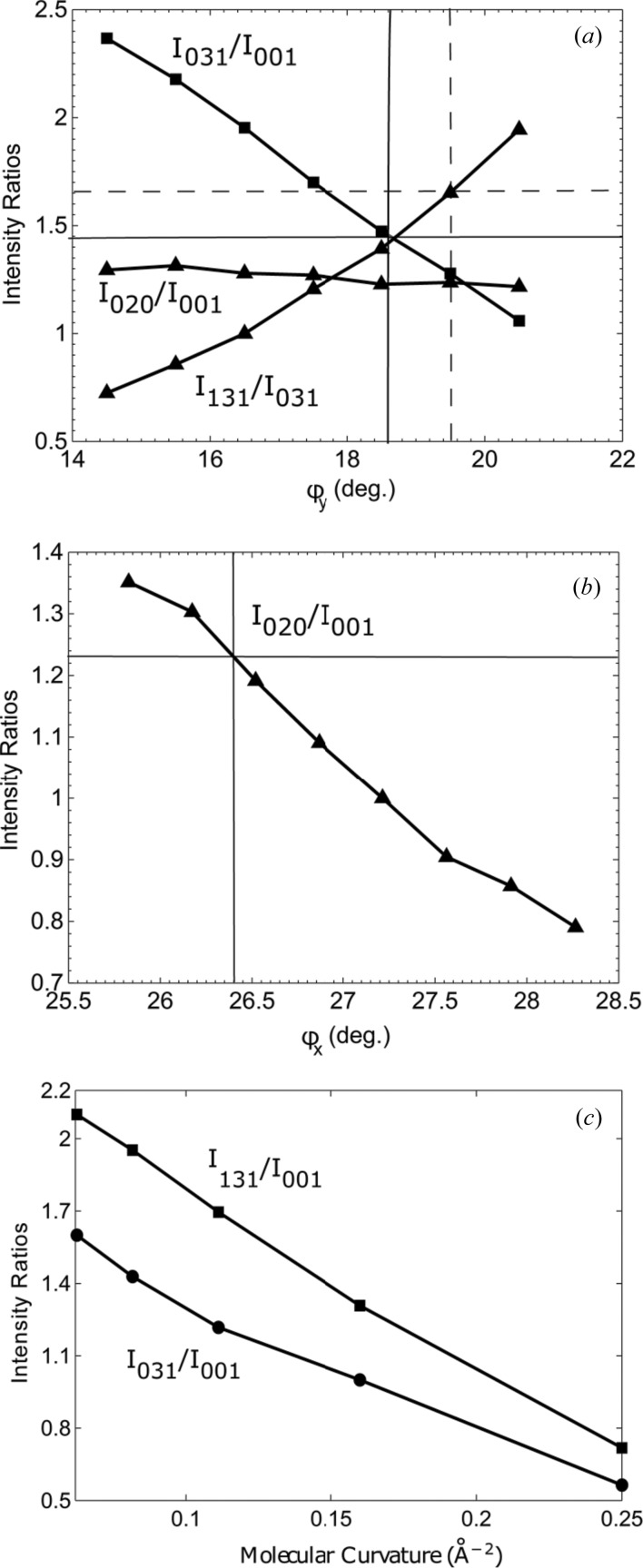
Variations of peak intensity ratios of selected reflections as functions of the (*a*) 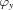 angle, where the vertical and horizontal lines refer to values of the intensity ratios (solid line) from Pagola *et al.* (2000 ▸) and the XFEL data (dashed line), and (*b*) 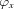 angle, where the vertical and horizontal lines refer to values of the intensity ratios from Pagola *et al.* (2000 ▸), and (*c*) the curvature of the molecule.

**Figure 4:**
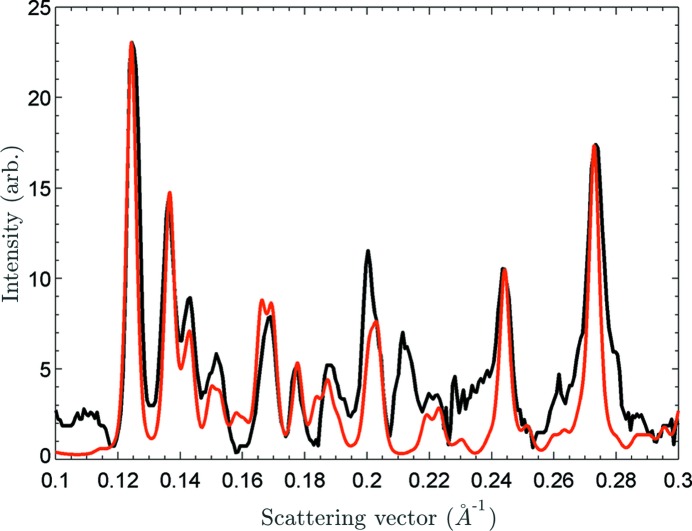
The measured XFEL data (black) compared with our model for β-hematin incorporating structural inhomogeneity (red).

Fig. 3 ▸(*a*) shows the sensitivity of the *I*_131_/*I*_031_ ratio to the tilting angle, φ*_y_*. We observe that the larger the tilt, the larger the ratio of peak intensities for the corresponding Bragg reflections. Meanwhile, the variation of angle φ*_x_* (see Fig. 2 ▸ *c*) affects the *I*_020_/*I*_001_ ratio (Fig. 3 ▸ *b*). The curvature of the FP—Fe^III^ molecule also affects the powder diffraction pattern. Fig. 3 ▸(*c*) shows the variation of the *I*_031_/*I*_001_ and *I*_131_/*I*_001_ ratios as a function of the curvature of the FP—Fe^III^ molecule. As one can see, the flatter the molecule, the stronger the 031 and 131 Bragg reflections with respect to the 001 Bragg reflection. By adjusting the translational vector, **Δ**, and the orientation of the FP dimer we were able to generate diffraction patterns that correspond to the XFEL XRPD pattern (Fig. 4 ▸) and were able to simulate the correct intensity distributions of most of the Bragg reflections. The generated intensity ratios of the selected reflections, *I*_020_/*I*_001_, *I*_131_/*I*_013_, *I*_031_/*I*_001_ and *I*_131_/*I*_001_, are in a good agreement with experimental data as shown in Fig. 4 ▸ (additional details of the model are included in the supplementary information).

However, there is also some inconsistency between the simulated and the measured intensity distributions, particularly within the 0.21–0.22 Å^−1^ range of the scattering vector. The discrepancy may arise from the fact that in our analysis we considered only one possible configuration of the FP dimer, while several configurations of FP dimer could be present within β-hematin crystals, as has been shown previously (Straasø *et al.*, 2011 ▸).

Different configurations affect the displacement vector **Δ** and, therefore, the packing of the FP dimers into the translational lattice. Moreover, we considered only the Fe1—O40 bonds formed by the Fe1 and O40 atoms (Fig. 2 ▸). The equivalent connection between two FP molecules can be achieved between the Fe1 and O37 atoms, which affects the direction and the magnitude of the displacement parameter Δ*_x_*. Thus, according to our analysis, the extra intensity in the 0.21–0.22 Å^−1^ region, and other more subtle differences observed around 0.16 Å^−1^ and in the 0.18–0.19 Å^−1^ range, is caused by the presence of crystals with various configurations of the FP dimers, which was not taken into account during the simulation of the diffraction pattern shown in Fig. 4 ▸. Another explanation for the discrepancy could be local radiation damage effects, which as discussed previously may have an influence on the relative intensity of diffraction peaks. The combination of these effects, structural and radiation induced, could also account for these differences.

## 3. Discussion   

From a simple model analysis of the effect of local radiation damage, under the conditions used here, the magnitude of the effect of ionization of Fe does not appear to account for the observed changes in the ratio of reflection intensities. However, given the complex interplay of local and global radiation damage effects, we emphasize that it is not possible, at present, to entirely discount the effect that radiation damage may have on the data. As an alternative explanation, models which include structural disorder appear to be able to reproduce the peak intensity ratios observed at the XFEL. This would indicate that nanocrystals of β-hematin have a different structure compared to the larger crystals. Recent molecular dynamics simulations using *CHARMM* (https://www.charmm.org/) in combination with EXAFS data (Kuter *et al.*, 2014 ▸) also indicate that in aqueous solution the solvation influences the ferriheme speciation and configuration (de Villiers *et al.*, 2007 ▸; Crespo *et al.*, 2010 ▸; Asher *et al.*, 2009 ▸). In addition, the conformational flexibility of the propionic side chains allows FP molecules to allocate the oxygen ions at the correct positions to form Fe—O and O—H⋯O connections with the nearest-neighbor molecules. These factors play an important role in exploring conformational space, and facilitating initial intermolecular interactions with Fe atoms in adjacent heme molecules with different spatial displacements and orientations. This flexibility probably nucleates the formation of various dimers and higher oligomers and stabilizes the early stage crystalline structure. During the growth process the solvation changes and reduces, facilitating formation of ‘correct’ hydrogen bonds between FP molecules. The flexibility of the propionic side chains thus decreases, providing increased periodicity in the crystal lattice resulting in a more ordered structure.

If confirmed, the structural disorder interpretation of our data has a number of implications for hemozoin detoxification and antimalarial drug action. While sequestration of FP into hemozoin reduces its toxicity, we previously showed that the large hemozoin crystals isolated from mature trophozoite stages still retain the ability to mediate oxidative damage, albeit at a rate ∼100-fold lower than non-crystallized FP (Klonis *et al.*, 2010 ▸). It is likely that small nascent crystals with increased structural inhomogeneity (and increased surface area) exhibit even higher pro-oxidant activity. Indeed, quinoline and related antimalarials, such as chloro­quine, mefloquine and lumefantrine, are thought to inhibit hemozoin formation by binding to both free FP–Fe^III^ and the surface of the hemozoin crystal, thus preventing the addition of further hematin molecules (Olafson *et al.*, 2015 ▸; Klonis *et al.*, 2010 ▸; Hanscheid *et al.*, 2007 ▸; Combrinck *et al.*, 2013 ▸). The predicted (fastest growing) surface of the macroscopic crystals has been used to design compounds that are expected to inhibit crystal growth (Buller *et al.*, 2002 ▸). Our data indicate that nascent crystals present previously unappreciated surface structures with different molecular orientations of the propionic acid side chains. These could be targeted for the design of novel antimalarials. For example, compounds that decrease the flexibility of the propionate side chains would considerably slow (or even prevent) the nucleation process.

Recent experimental studies of the interaction of chloro­quine and ferriheme in aqueous solution show that chloro­quine binds ferriheme in the μ-oxo dimeric form (Kuter *et al.*, 2014 ▸). Our data may suggest that quinoline compounds in which the amino side chains form a very tight interaction with the propionate side chains of FP should decrease the flexibility of these chains. Docking of chloro­quine between two porphyrin rings of the μ-oxo dimer formed on the surface of nanocrystals could then apply the most rigid constraint to the flexibility of the propionate side chains. This would prevent the propionate side chains from exploring the conformational space needed for the formation of dimers and higher oligomers, which is likely to be critical to the early stages of hemozoin formation. One possibility is to design aminoquinolines that are optimized to interact with the propionate groups of adjacent FP molecules at the surface of nascent crystals.

## 4. Summary and conclusions   

In summary, our results show how serial femtosecond nanocrystallography can produce information from nanocrystals of β-hematin that is markedly different from data collected at the synchrotron. If the structural interpretation of these differences proves correct, the results point to nanocrystallography as being able to provide information about conformational states that are not accessible using conventional crystallography, providing fundamentally new opportunities for drug discovery. We have applied this idea to investigate the early stages of crystallization of hemozoin through structural studies of its synthetic phase, β-hematin. Our data suggest that, when quinoline compounds form a very tight interaction with the propionate side chains of FP–Fe^III^, the flexibility of these chains is decreased. This would prevent them from exploring the conformational space needed for the formation of dimers and higher oligomers, which is important in the early stages of hemozoin formation and thus critical for the survival of the malaria parasite.

## 5. Materials and methods   

### 5.1. β-Hematin preparation   

β-Hematin crystals were prepared in an aqueous phase using the method described by Jaramillo *et al.* (2009 ▸). In this method, 50 mg of hematin was solubilized in 8.6 ml of 70 m*M* NaOH before being acidified by the dropwise addition of 2.9 ml glacial acetic acid. The sample was then incubated for 18 h at 343 K to allow formation of β-hematin and subjected to the following washing regimen: (i) three 3 h incubations in 0.1 *M* NaHCO_3_ on a rotator with three washes in water prior to each addition of fresh NaHCO_3_; (ii) three washes in methanol with three washes in water prior to each addition of fresh methanol. The sample was then washed twice with water and stored at 277 K.

The preparation used to generate the crystals (Jaramillo *et al.*, 2009 ▸) was selected because of the ease with which it is possible to produce sufficient sample for SFX measurements. However, in previous studies this protocol has been found to produce lower-quality crystals composed of smaller crystallites than the naturally derived hemozoin product (Kapishnikov *et al.*, 2012 ▸). To test the validity of this protocol as a model system for investigating hemozoin, the crystallite sizes and interatomic distances and angles derived from synchrotron diffraction data for these samples were compared to results previously published for naturally derived hemozoin. The data for the unfiltered sample (MX1) were found to give a very close match to the natural hemozoin. In the case of the filtered sample (MX2) the data were also comparable, with the exception of the calculated |Fe1—O40| bond length where significant differences were observed. We also compared the crystallite size obtained from the MX1 and MX2 experiments, based on the FWHM of the 001 reflection, and found that the β-hematin preparations produced crystallites of around 50% the size of the natural hemozoin (see supplementary data). For the present work investigating the early stages of hemozoin formation we concluded that the system measured was an adequate biomimetic model of the natural product. In the case of larger crystals, however, we note that the preparation used to produce the β-hematin would not be an adequate model for the natural hemozoin.

### 5.2. Laboratory measurements   

The size of crystals before and after filtering was determined using a JEOL JEM-2010 transmission electron microscope.

### 5.3. Data collection   

XFEL diffraction data were collected at the LCLS coherent X-ray imaging (CXI) beamline with a peak fluence of 7.5 × 10^11^ photons per 30 fs pulse. We used an 8.5 keV (1.46 Å wavelength) X-ray beam, focused to an area of 1 × 1 µm (FWHM). Samples were injected into the sample-beam interaction region inside a vacuum chamber (Boutet & Williams, 2010 ▸) using a gas dynamic virtual nozzle injector (Weierstall *et al.*, 2012 ▸; DePonte *et al.*, 2008 ▸). The very bright micro-focus XFEL source allows us to collect diffraction patterns from individual β-hematin nanoscale crystals. Owing to the polycrystalline nature of each crystal, partial powder rings were recorded with each shot. Single-pulse diffraction patterns from β-hematin nanocrystals were recorded on a CSPAD (Hart *et al.*, 2012 ▸) containing 1516 × 1516 square pixels, each of area 110 µm^2^. β-Hematin nanocrystals were delivered in a solution of ultrapure water.

Over 60 000 single-pulse two-dimensional diffraction patterns were analyzed. More than 90% of diffraction patterns collected contained data from β-hematin, as determined by the detection of low-resolution powder rings where the background was low. A detector dark calibration was performed and any bad pixels masked. The two-dimensional diffraction patterns were analyzed individually by *FIT2D* (Hammersley, 2016 ▸; Hammersley & Riekel, 1989 ▸) to produce radially averaged one-dimensional XRPD patterns of β-hematin. The Rietveld refinement was performed using *RIATAN-2000* (Hammersley & Riekel, 1989 ▸) and maximum entropy analyses using *PRIMA* (Izumi & Dilanian, 2002 ▸). The split pseudo-Voigt function of Toraya (1990 ▸) was used as a profile function. The background was represented by a composite background function [*i.e.* an 11th-order Legendre polynomial multiplied by a set of numerical values obtained with *PowderX* (Dong, 1999 ▸) to approximate the background]. Coefficients for the analytical approximation to atomic scattering factors for Fe, O, N and C were taken from Wilson (1992 ▸). Anomalous scattering factors were taken from Kissel & Pratt (1990 ▸). The unit-cell parameters were determined using *DICVOL* (Boultif & Louer, 2004 ▸), and further refined by *RIETAN-2000* (Hammersley & Riekel, 1989 ▸). The effect of the preferential orientation of crystallites on the XRPD pattern of β-hematin was corrected for using the March–Dollase function (Dollase, 1986 ▸). Data presented in the paper were scaled to the first (most intense) peak in the data (scattering vector ∼0.13 Å^−1^).

Synchrotron X-ray diffraction data were collected at beamlines MX1 and MX2 at the Australian Synchrotron. We used a 13.0 keV (0.95 Å wavelength) X-ray beam with an incident flux of 1.4 × 10^11^ photons s^−1^ and 1 × 10^12^ photons s^−1^ for MX1 and MX2, respectively. The sample was mounted on the micromesh holder and frozen under a liquid nitro­gen stream with 30% glycerol as a cryoprotectant. The diffraction pattern was detected using a CCD camera containing a 2048 × 2048 array of 102.4 µm pixels and a 3072 × 3072 array of 102.6 µm pixels at the MX1 and MX2 beamlines, respectively.

## Supplementary Material

Additional experimental data are shown in Figs. S2 and S3,;additional simulation data iareshown in Fig. S4. DOI: 10.1107/S1600576717012663/ap5010sup1.pdf
