# Supplementary material for: Nanocrystallography measurements of early stage synthetic malaria pigment
Source: J Appl Crystallogr. 2017 Sep 28;50(Pt 5):1533–40. doi: 10.1107/S1600576717012663 (PMC5627683; doi:10.1107/S1600576717012663)
Supplement: Supplementary file 1 [file j-50-01533-sup1.pdf]

JOURNAL OF APPLIED CRYSTALLOGRAPHY

Volume 50 (2017)

Supporting information for article:

Nanocrystallography measurements of early stage synthetic malaria pigment

Ruben A. Dilanian, Victor Streltsov, Hannah D. Coughlan, Harry M. Quiney, Andrew V. Martin, Nectarios Klonis, Con Dogovski, Sébastien Boutet, Marc Messerschmidt, Garth J. Williams, Sophie Williams, Nicholas W. Phillips, Keith A. Nugent, Leann Tilley and Brian Abbey

Supplementary information: **Nanocrystallography provides insights into the growth, structure and disorder of early stage synthetic malaria pigment**

Table S1. Inter-atomic distances and angles.

	hemozoin[a]	β-hematin Synchrotron data (MX1)	β-hematin Synchrotron data (MX2)
|Fe1 – O40|	1886(2) Å	1.91(2) Å	2.51(8) Å
|Fe1 – Fe2|	8.01(6) Å	7.93(5) Å	8.42(7) Å
|Fe1 – Fe3|[b]	9.07(6) Å	9.17(6) Å	9.51(6) Å
|Fe1 – Fe4|[c]	8.02(6) Å	8.01(6) Å	8.05(6) Å
F2 – F1 – F4	111.62°	112.19°	109.48°
F1 – F2 – F3	68.38°	67.81°	70.52°

[(a)]Data from Klonis N. *et al*., Biochemistry, **49**, 6804-6811 (2010)

[(b)]β–Pr; [(c)]P-type

Additional model details

We assume the FP–Fe(III) molecule is in an orthogonal system and that the porphyrin ring of the molecule is located in a (xy) plane (Figure S1). Given the orientation the Fe–O bonds, which are normal to the porphyrin ring planes, and the rigid structure of the porphyrin ring of the FP–Fe(III) molecule, the relative displacement of two FP–Fe(III) molecules forming a FP–Fe(III) dimer can be described by the displacement vector, $\boldsymbol{\Delta} = (\Delta_X, \Delta_Y, \Delta_Z)$, where

$$\begin{aligned} \Delta_X &= |x_{Fe}| + |x_O| \\ \Delta_Y &= |y_{Fe}| + |y_O| \\ \Delta_Z &= |z_{Fe}| + |z_O| + d_{|Fe-O|} \end{aligned}$$

(x_{Fe}, y_{Fe}, z_{Fe}) and (x_O, y_O, z_O) are coordinates of the Fe and the O atoms forming the Fe–O bond, respectively, and $d_{|Fe-O|}$ is the length of the Fe–O bond. The relative displacement of the molecules is defined by the relative displacement of the Fe atom from the porphyrin ring plane and by the position of the O atom. The latter is influenced by the conformation of the propionate side chain.

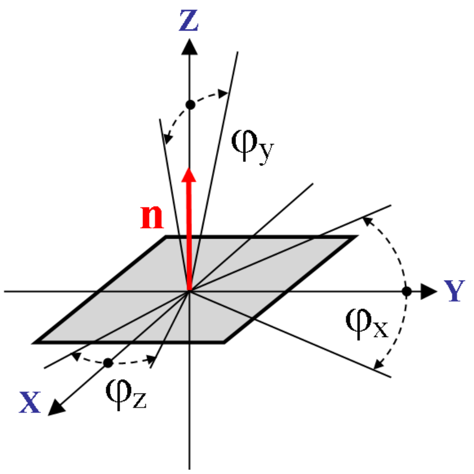

Figure S1. Coordinate system used to define the geometry and orientation of porphyrin ring (represented by the grey square).

Each FP–Fe(III) dimer contributes its vinyl and propionate ends to interactions with two different FP–Fe(III) dimers to form extended chains in a polymeric interaction. This interaction involves contacts between the noncoordinated face of the propionate end of one dimer and the coordinated face and vinyl region of the other. The dimers have the same orientation but are translated along a vector, $\mathbf{T_1}$ (figure 2d), which corresponds to the [001] crystallographic direction, the longest dimension of the β-hematin crystal. Chains form two-dimensional layers through the π-π interaction face (Klonis *et al.*, 2010). The formation of these layers can be described by translation along a vector, $\mathbf{T_2}$ (figure 2d), which corresponds to the [010] crystallographic direction. The porphyrin rings of two neighboring molecules, belonging to two different P-type chains, are arranged in such a way that their noncoordinated faces are mutually opposed. The considerable spatial overlap of these porphyrin rings defines the π-π interaction between the two FP–Fe(III) molecules. The β-hematin crystal, therefore, can be described as a series of two-dimensional layers of FP–Fe(III) dimers translated the [100] crystallographic direction, the smallest dimension of the β-hematin crystal. The two-dimensional layers are interlinked by O–H···O bonds between two propionate side chains, CH_3–CH_2–C(=O)–O, of two neighboring FP–Fe(III) molecules belonging to two different layers.

The orientation of the FP–Fe(III) dimer is described via the displacement vector, $\mathbf{\Delta}$. The porphyrin rings of the FP–Fe(III) molecules are oriented close to the (031) and (131) crystallographic planes. The ratio of intensities of the (031) and (131) reflections is determined by the proximity of these crystallographic planes to the plane of the porphyrin rings. The orientation of the porphyrin rings with respect to these crystallographic planes is defined by the φ_Y angle.

Given the atomic coordinates of a single FP–Fe(III) molecule (Ref[8]), the spatial and the orientational alignment of other FP–Fe(III) molecules within the three-dimensional periodic lattice can be described via displacement and

translational vectors and, in turn, a corresponding diffraction pattern can be generated. We find that the relative displacement of two molecules within the dimer plays a key role in the formation of the diffraction pattern.

Using the published atomic coordinates for β-hematin [8] we refined the displacement vector and the orientation of the FP–Fe(III) dimer to simulate diffraction patterns with various ratios of peak intensities of selected Bragg reflections. We observe that the flatter the molecule, the stronger the (031) and the (131) Bragg reflections, with respect to the (001) Bragg reflection. The notable drop of the peak intensities of (031) and (131) Bragg reflections, observed in the XFEL powder diffraction pattern can by reproduced by adjusting the curvature of the FP–Fe(III) molecules within the β-hematin nanocrystals (Figure 4).

Table S2. Full-width at half maximums of the (001) reflection and the corresponding crystallite size.

Sample	FWHM (deg.)	Crystallite size (nm)
MX1 (unfiltered)	0.224	25
MX2 (filtered)	0.184	30
Hemozoin[a]	0.077	71

[(a)]Data from Klonis N. *et al*., Biochemistry, **49**, 6804-6811 (2010)

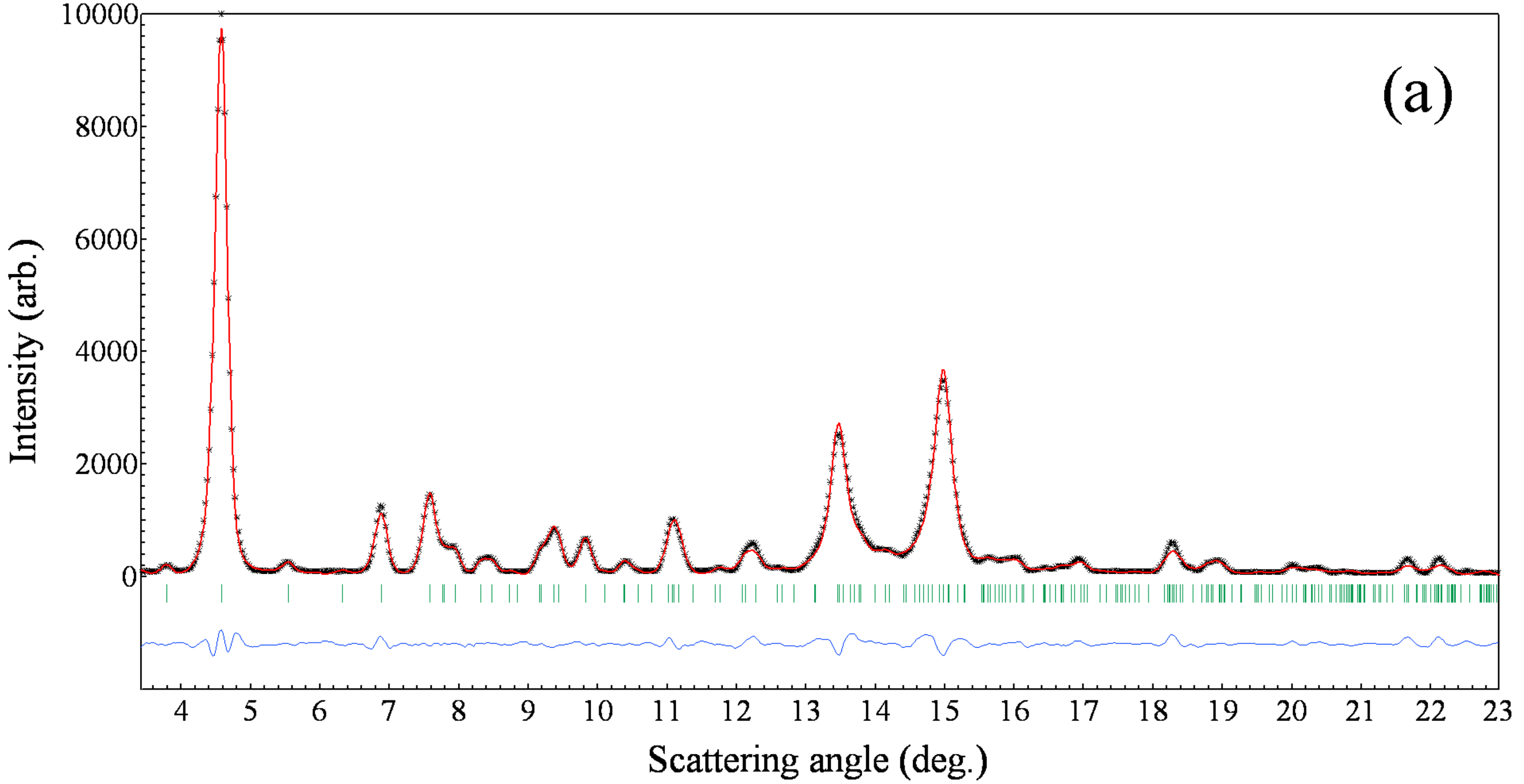

Figure S2: Rietveld-refinement patterns of β-hematin. The red lines are calculated intensities, the black markers are observed intensities, blue lines are differences between calculated and measured intensities, green vertical lines indicate peak positions of Bragg reflections. Synchrotron data are consistent with published structure of β-hematin and similar to structure of hemozoin (see Table S1).

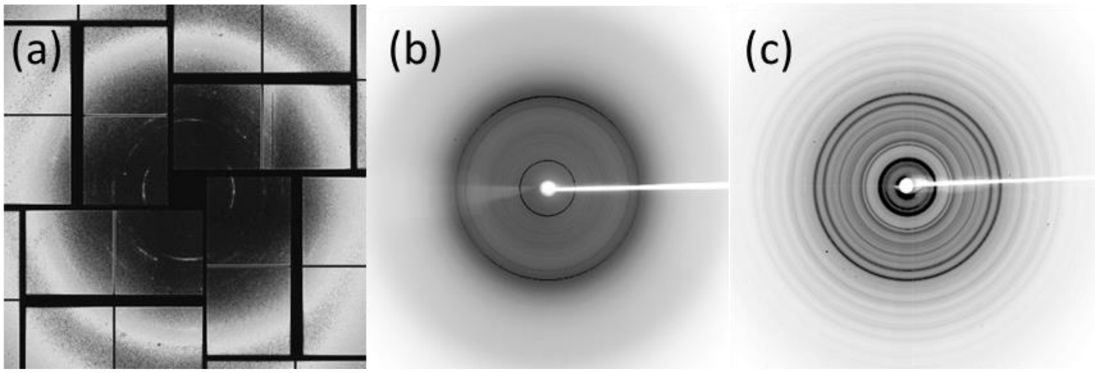

Figure S3 Single frames of raw, unprocessed data from the (a) XFEL (corresponding to region spanned by data), (b) MX2 and (c) MX1.

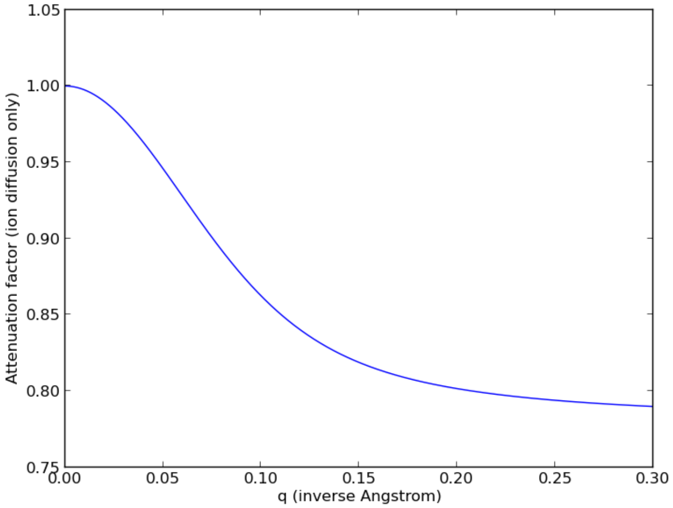

Figure S4. Dynamic attenuation factor for β-hematin, assuming a 1 × 1 μm beam at 8.5 keV and 30 fs pulse duration with a peak fluence of 7.5×10^{11} photons per pulse.
